# Splenic Injury as a Complication of Robotic-Assisted Thoracic Surgery

**DOI:** 10.1093/icvts/ivag096

**Published:** 2026-04-07

**Authors:** Ho Seong Cho, Jeong Su Cho, Hyo Yeong Ahn, Yeong Dae Kim

**Affiliations:** Department of Thoracic and Cardiovascular Surgery, Biomedical Research Institute, Pusan National University Hospital, School of Medicine, Pusan National University, Busan 50612, South Korea; Department of Thoracic and Cardiovascular Surgery, Biomedical Research Institute, Pusan National University Hospital, School of Medicine, Pusan National University, Busan 50612, South Korea; Department of Thoracic and Cardiovascular Surgery, Biomedical Research Institute, Pusan National University Hospital, School of Medicine, Pusan National University, Busan 50612, South Korea; Department of Thoracic and Cardiovascular Surgery, Biomedical Research Institute, Pusan National University Hospital, School of Medicine, Pusan National University, Busan 50612, South Korea

**Keywords:** robotic-assisted thoracic surgery, splenic injury, splenectomy, lobectomy

## Abstract

Splenic injury after thoracic surgery is rare and has not been reported following robotic-assisted thoracic surgery (RATS). We describe a 52-year-old woman who underwent RATS left upper lobectomy. After discharge, she returned with abdominal pain and hypotension. Computed tomography revealed hemoperitoneum with signs of splenic injury. Emergent laparoscopic splenectomy confirmed splenic rupture. This case highlights that unrecognized intraabdominal trauma can occur during RATS; surgeons should exercise caution near the diaphragm and remain vigilant post-discharge.

## INTRODUCTION

Splenic injury following video-assisted thoracic surgery (VATS) is rare, with only a few cases reported in the literature.^1^ However, splenic injury has been more frequently described as a complication of blind chest tube insertion.^2^ Although splenic injury can also occur after robotic-assisted thoracic surgery (RATS), to our knowledge, no specific reports have documented it. Herein, we report a case of splenic injury after RATS lobectomy, diagnosed after discharge, and treated by laparoscopic splenectomy.

## CASE

**Figure 1.**
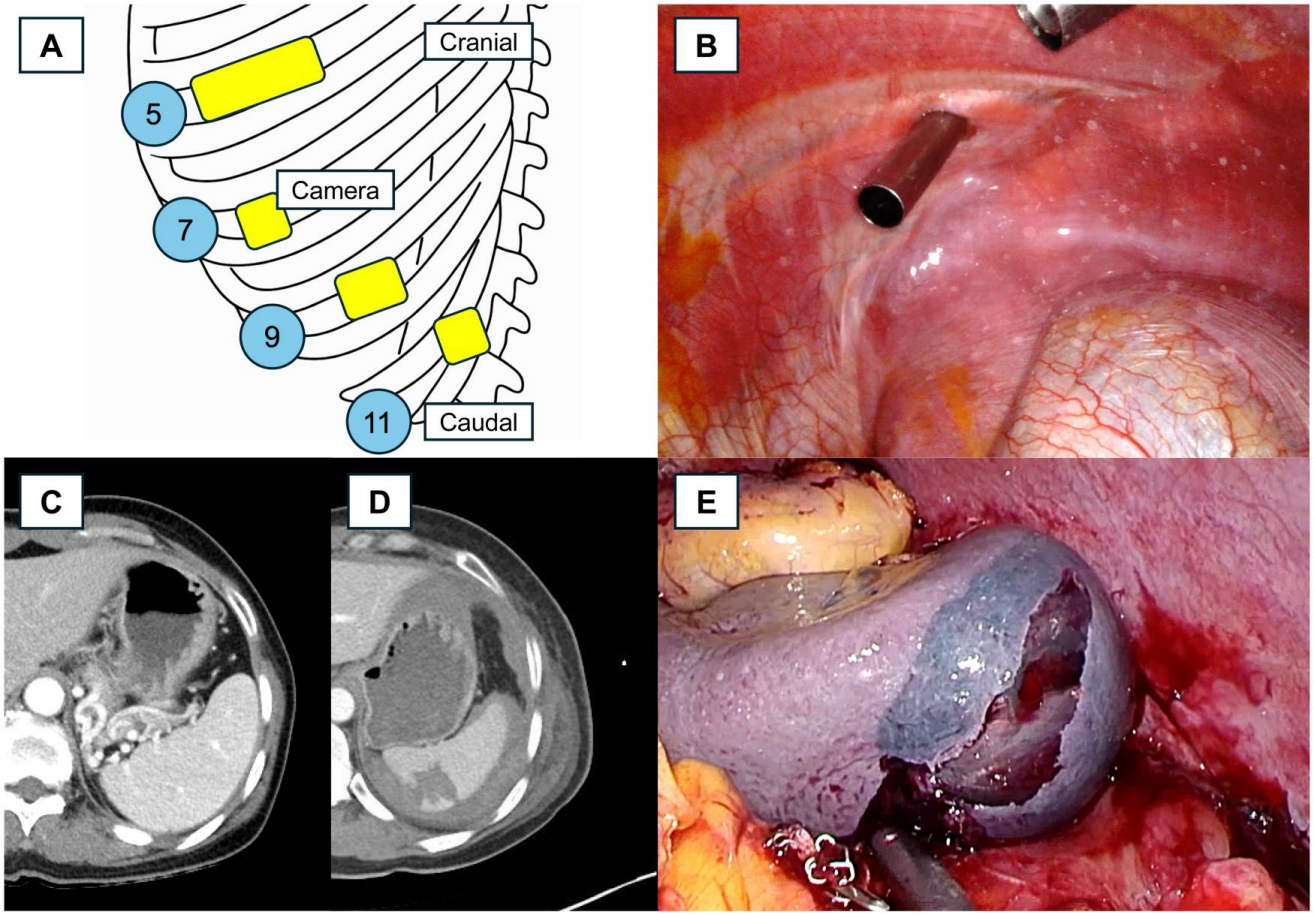
(A) Schematic of Port Placement for Anterolateral RATS Lobectomy. (B) Relationship Between Ports and the Left Diaphragm. (C) Preoperative CT Showing a Normal Spleen. (D) CT Showing a Low-Density Splenic Lesion with a Sentinel Clot Sign Around the Spleen, Indicating Recent Bleeding from Splenic Injury. (E) Intraoperative Image Showing the Splenic Injury Site

A 52-year-old woman was admitted with a 24-mm ground-glass nodule in the left upper lobe. We planned diagnostic wedge resection and, if malignancy was confirmed, left upper lobectomy with mediastinal lymph node dissection. A RATS approach was selected using the da Vinci Xi system (Intuitive Surgical). Following the anterolateral RATS technique described by Kang et al., to enable a 4-arm configuration, an initial utility window was created at the fifth intercostal space, CO_2_ insufflation was initiated and maintained at 8 mmHg, and additional ports were sequentially placed under thoracoscopic vision at the 7th, 9th, and 11th intercostal spaces (Figure 1A and B, Video 1).^3^ No pleural adhesions or intraoperative difficulties were encountered. Frozen section analysis revealed adenocarcinoma, and the left upper lobectomy was therefore performed. Specimen extraction was performed through the utility window. During this step, the remaining docked robotic arms were fully retracted and parked laterally close to the chest wall to minimize inadvertent contact. Mediastinal lymph node dissection was completed, including stations 4 l, 5, 6, 7, 8 l, and 9 l. No intentional downwards retraction of the diaphragm was required under CO_2_ insufflation, and no diaphragmatic injury was noted intraoperatively. The operation was uneventful, and the patient was discharged on postoperative day (POD) 5. No abdominal pain was reported before discharge, and laboratory tests and postoperative chest X-ray were unremarkable. Final pathology revealed adenocarcinoma with pT1bN0.

The day after discharge, the patient presented to the emergency department with abdominal pain and dizziness. Initial evaluation revealed severe hypotension and tachycardia. Following resuscitation, computed tomography (CT) of the thorax and abdomen demonstrated hemoperitoneum surrounding the spleen, with a sentinel clot sign suggestive of splenic injury (Figure 1D). An emergent laparoscopic splenectomy was performed (Figure 1E). Intraoperative findings confirmed splenic rupture without diaphragmatic injury. The patient’s condition stabilized postoperatively, and she was discharged on POD 8 without complications (Table 1). Pathologic examination of the spleen revealed no specific abnormalities except capsular laceration.

**Table 1.** Perioperative Vital Signs and Laboratory Test Results

	Admission	POD 0	POD 1	POD 3	POD 5	POD 6 (ER visit)	Post splenectomy
**BP, mmHg**	120/60	153/103	116/59	136/88	137/84	60/-	123/81
**HR, bpm**	80	63	83	84	83	98	81
**Hb, g/dl**	12.6	12.2	10.6	9.1	9.1	8.3	8.8
**Hct, %**	37.1	35.4	30.8	25.1	26.7	23.7	25.3
**Plt, 10^3^/µL**	167	155	123	118	129	200	148

Abbreviations: BP, blood pressure; ER, emergency room; Hb, haemoglobin; Hct, hematocrit; HR, heart rate; Plt, platelet; POD, postoperative day.

## DISCUSSION

Although VATS was the predominant minimally invasive approach, robotic lobectomy is now increasingly adopted by thoracic surgeons. Compared to VATS or open surgery, RATS may entail a higher risk of unintentional intraabdominal injury because of the lack of tactile feedback at the robotic console. Additionally, bleeding from such injuries may not be visible within the thoracic field, rendering intraoperative detection difficult. Consequently, injuries such as the splenic rupture in this case may remain undiagnosed until recovery or clinical deterioration.

The anterolateral RATS technique described by Kang et al. offers several advantages. Using 4 robotic arms minimizes the need for an assistant, facilitating solo surgery.^3^ However, caution is advised when placing ports near the 11th intercostal space, because of its proximity to the diaphragm and intraabdominal organs. Inadvertent traction may transmit force across the diaphragm and predispose to occult splenic injury. As preventive measures, we now place the most caudal port at the 10th intercostal space along the posterior axillary line and minimize robotic arm motion during transient CO_2_ loss, such as specimen extraction, to reduce inadvertent caudal traction or torque across the left hemidiaphragm.

In this case, the patient’s laboratory results and vital signs during hospitalization were nonspecific, suggesting that the splenic injury went undetected. It is likely that the spleen sustained an occult, non-penetrating contact injury from the robotic arm during the operation and subsequently ruptured during routine activities after discharge. We suspect that this may have occurred during transient loss of CO_2_ insufflation when the diaphragm rises, and the spleen becomes more vulnerable to transmitted traction or torque across the left hemidiaphragm. This clinical course resembles delayed splenic rupture following blunt trauma. Clinicians should remain vigilant for this possibility in patients presenting with unexplained hypotension or abdominal symptoms.^4^

In conclusion, splenic injury can occur during RATS even in the absence of diaphragmatic disruption. Surgeons should exercise caution when operating near the diaphragm to avoid inadvertent injury to intraabdominal structures.

## Data Availability

Available on reasonable request.
